# Parkinsonism in a Patient With Metal on Metal Total Hip Replacement Related Elevated Serum Heavy Metal Levels

**DOI:** 10.7759/cureus.17791

**Published:** 2021-09-07

**Authors:** Charlotte Lee, Michele Hu

**Affiliations:** 1 Oxford University Medical School, Medical Sciences Division, University of Oxford, Oxford, GBR; 2 Oxford Parkinson's Disease Centre, University of Oxford, Oxford, GBR; 3 Nuffield Department of Clinical Neurosciences, Medical Sciences Division, University of Oxford, Oxford, GBR

**Keywords:** parkinsonian presentation, chromium, cobalt, heavy metal poisoning, arthroplasty/replacement, parkinson's disease

## Abstract

Heavy metal toxicity accounts for a rare minority of parkinsonian presentations. It is established that metal on metal total hip replacements can elevate serum cobalt and chromium levels. Here, we present an atypical parkinsonism presentation in a patient with metal on metal total hip replacement related heavy metal exposure, a picture complicated by a previous cerebral vascular accident involving the right lentiform nucleus. Initial work-up included MRI brain and dopamine transporter uptake imaging. A follow-up consultation revealed progressing parkinsonism. This case presentation reports the first parkinsonism presentation associated with metal on metal total hip replacement related heavy metal toxicity.

## Introduction

Parkinson’s disease (PD) is a chronic, progressive disorder of somatic motor function, primarily affecting the basal ganglia [1]. Pathogenesis is complex, involving α-synuclein fibril and oligomer formation [1]. Parkinsonism describes a syndrome of distinct symptoms, including tremor, muscular rigidity, and bradykinesia, which can be caused by PD, trauma, infection, toxins, or iatrogenic [1].

The majority (80-90%) of PD is idiopathic [1]; however, a strong association with heavy metal toxicity has previously been demonstrated, predominantly reported after pesticide exposure or water supply contamination [2]. Copper, iron, and cobalt toxicity are known causes of parkinsonism [3]. Further, it is known that in situ metal on metal joint replacements can cause elevated heavy metal serum levels [4]. Determination of PD/parkinsonism aetiology is important to predict prognosis and can affect management. Associations between PD and mental health diagnoses are well established [5]. Determining disease aetiology can have significant psychological benefits, especially if patients have known exposure to risk factors.

Previous epidemiological studies have demonstrated an association between elevated serum cobalt levels and cognitive decline, peripheral neuropathy, and sensorineural hearing loss [6]. Despite the demonstration of cobalt causing significant acceleration in α-synuclein fibril formation, there are no reports of elevated cobalt serum levels related to in situ joint replacements and parkinsonism [7]. Here, we discuss a patient presenting with parkinsonian features, with a history of recent cerebrovascular accident and elevated cobalt and chromium serum levels related to a metal on metal total hip replacement (THR).

## Case presentation

**Figure 1:**
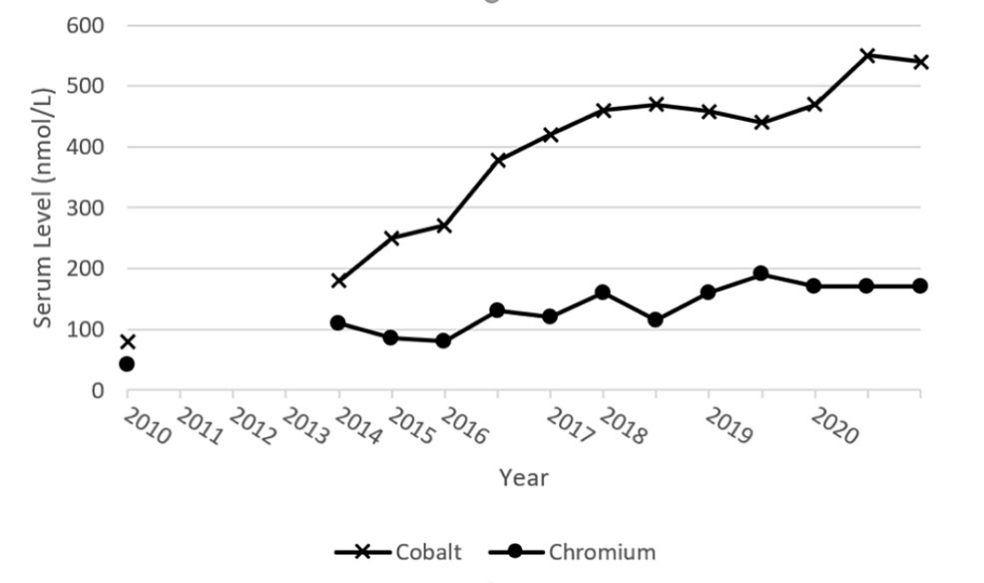
Patient serum cobalt and chromium levels (2010-2020). Normal serum reference ranges: cobalt 0-20 nmol/L and chromium 0-100 nmol/L

An 84-year-old right-handed man presented to clinic with bradykinesia and gait instability. His past medical history included a cerebral vascular accident (CVA) affecting the right middle cerebral artery (MCA) territory infarct involving the right basal ganglia and insula, five months prior to presentation. This CVA clinically manifested as left-sided weakness, slurred speech, and eye deviation. Atrial flutter was diagnosed and the patient was commenced on apixaban (5mg BDS). CT confirmed decreased MCA hyperintensity in response to thrombolysis and the patient experienced good functional recovery; however, with residual reduction in fine motor dexterity. The patient underwent a right metal on metal THR in 2005 and is under ongoing monitoring for increasingly high serum cobalt and chromium levels related to this metal implant (Figure 1). There was no previous significant medical history. The patient reported a stable weight and BMI was calculated to be 23. Drug history included only apixaban 5mg BDS.

On examination, the patient exhibited marked axial rigidity and mild left ankle rigidity. Foot tapping demonstrated significant left foot decrement in comparison with right foot. This asymmetricity was reproduced with finger tapping. The patient demonstrated a shuffling gait, difficulty turning, and a positive pull test. Examination of eye movements elicited no positive neurological signs. There was no tremor, no non-motor PD features, and no change in cognition. Frontalis overactivity was noted, with difficulty to complete the Luria test, however, applause sign was negative. Manual dexterity was decreased bilaterally. Handwriting was unaffected. The patient reported his speech was quieter, and dribbling from the left-hand side of his mouth.

Six months post initial presentation, the patient was commenced on co-careldopa due to symptom progression, gait freezing, and a history of fall. There were possible ‘round the house’ movements on examination of eye movements. Difficulties completing the Luria test remained, however, the applause sign remained negative. Given these atypical features and clinical suspicion of early progressive supranuclear palsy, PD diagnosis was not confirmed and a six-month review is currently scheduled. The patient continues to undergo review of their heavy metal serum levels. Currently, these have plateaued (Figure 1). Given this, and the novel association of metal on metal THR-related heavy metal exposure and parkinsonism, therefore, the uncertain contribution of exposure to disease, the patient’s functional THR remains in situ.

Imaging studies

As cobalt and chromium are both ferromagnetic, magnetic resonance (MR) imaging was undertaken to potentially visualise heavy metal deposition in basal ganglia structure. MR imaging is sensitive to cobalt. Furthermore, MR imaging would enable characterisation of patient neuroanatomy (especially with regard to the previous right MCA infarct). MR imaging demonstrated a small serpiginous hemosiderin stained lacuna located in the right lentiform nucleus associated with peripheral increased T2-weighted-fluid-attenuated inversion recovery (T2/FLAIR) signal, consistent with the previous right MCA territory infarct. There was mild background small vessel disease, and notably, small for age basal ganglia. No heavy metal deposition was visualised.

To further define disease aetiology, brain ioflupane scan (DaTscan) was completed to characterise basal ganglia dopamine transporter activity six months after the first presentation to clinic. Decreased specific DaTscan uptake is consistent with dopaminergic synaptic loss. Patient imaging demonstrated a severe reduction in specific uptake of DaTscan within the right lentiform nucleus, particularly in the posterior aspect of the lentiform nucleus. Further, there was moderate reduction in the right head of the caudate. There was moderate reduction in DaTscan uptake within the posterior aspect of the left lentiform nucleus, consistent with early dopaminergic synaptic loss. There was good uptake in the left head of caudate for age.

## Discussion

It is well known that heavy metals play a vital role in a variety of physiological processes, for example, cobalt is a constituent of vitamin B12, deficiency of which can lead to subacute combined degeneration of the spinal cord [3]. Contrastingly, over-exposure to heavy metals and toxicity can be detrimental to health. As aforementioned, iron toxicity has been associated with parkinsonism and observed in degenerative dopaminergic neurons [3]. Copper has been demonstrated to promote α-synuclein aggregation [3]. The role of cobalt has not been sufficiently described in the literature, however, a role of reactive oxygen species has been suggested [3]. Here, we have described a patient with a history of right lentiform CVA and chronic cobalt toxicity, presenting with parkinsonism.

The complex picture of right lentiform nucleus historic infarct on bilateral lentiform dopaminergic degeneration in a patient exposed to heavy metals makes aetiological determination of the patient’s parkinsonian difficult. The patient’s symptom progression and bilateral disease imply a more classical dopaminergic neuronal degeneration-driven disease. Visually comparing the MR imaging with DaTscan imaging results overall suggested typical neurodegeneration of the left lentiform nucleus. The decreased DaTscan uptake seen on the right lentiform nucleus was in a similar anatomical area to the patient’s CVA. As such, it was concluded that both non-CVA neurodegenerative and CVA-related ischaemic changes were present and contributing to the clinical picture.

Besides direct microscopy studies, there are no current methods to distinguish neurodegenerative from heavy metal parkinsonism. Elevation of heavy metal serum levels following metal on metal hip replacements is well documented [4] and is associated with neurological disease, such as cognitive impairment and peripheral neuropathies [6]. Increasingly, evidence implicates the role of cobalt-derived free radical driven neurodegeneration and DNA damage in the basal ganglia, leading to PD/parkinsonism [3]. Furthermore, cobalt has been demonstrated to induce α-synuclein fibril formation [8]. However, there is currently insufficient evidence directly supporting a causal role of joint replacement-related cobalt in parkinsonism manifestation.

In addition to debated pathogenicity, the threshold heavy metal exposure for clinical significance is unclear [9]. It is unknown if a threshold serum level should dictate treatment, or clinical symptoms [9]. However, regardless of the precise threshold for consideration, our patient’s recorded serum cobalt levels since 2016 have exceeded all estimated thresholds that the authors recommended. Here, MR imaging was utilised to establish the deposition of heavy metals in the patient’s brain. Initially, imaging did not support a contributing role of heavy metal exposure in the patient’s presentation; the visual detection of cobalt deposition in the patient’s basal ganglia with MR imaging was negative. However, there is no reported evidence stating MR visualisation of cobalt as a threshold for determining pathological potential.

Despite lack of clear association between cobalt serum levels and parkinsonism, a recent MR imaging study supports cobalt as a contributing factor to this patient’s parkinsonism pathogenesis. In this study, patients with elevated serum cobalt from metal on metal hip replacements were shown to have more attenuated basal ganglia than those with non-heavy metal joint replacements [10]. Our patient’s small for age basal ganglia noted in MR imaging is compatible with this conclusion. Lack of serial imaging prevents study of our patient’s basal ganglia attenuation and serum cobalt levels.

## Conclusions

In summary, we report a case of parkinsonism in a patient with multiple factors potentially contributing to clinical manifestation, historic lentiform CVA and chronic heavy metal toxicity. Incidence of parkinsonism/PD in patients with metal on metal THR-related elevated heavy metal serum levels appears low but is not well described due to the ill-defined pathogenic role of cobalt. It is important to consider all potential contributing factors to clinical presentation, including in situ metal on metal joint replacements, for appropriate management of these patients. 
